# Prevalence of Frailty in European Emergency Departments (FEED): an international flash mob study

**DOI:** 10.1007/s41999-023-00926-3

**Published:** 2024-02-10

**Authors:** European Taskforce on Geriatric Emergency Medicine (ETGEM) collaborators, Timothy Coats, Simon Conroy, Bas de Groot, Pieter Heeren, Stephen Lim, Jacinta Lucke, Simon Mooijaart, Christian H. Nickel, Rose Penfold, Katrin Singler, James D. van Oppen, Effie Polyzogopoulou, Arina Kruis, Rosa McNamara, Bas de Groot, Santiago Castejon-Hernandez, Oscar Miro, Mehmet Akif Karamercan, Zerrin Defne Dündar, James D. van Oppen, Martina Pavletić, Pavla Libicherová, Frédéric Balen, Axel Benhamed, Xavier Dubucs, Romain Hernu, Said Laribi, Katrin Singler, Othon Fraidakis, Varvara Polyvios Fyntanidou, Effie Polyzogopoulou, Szabolcs Gaal, Anna Björg Jónsdóttir, Mary Elizabeth Kelly-Friel, Claire Alexandra McAteer, Lisa Diandra Sibthorpe, Aoife Synnott, Maria Beatrice Zazzara, Sophie Maria Coffeng, Bas de Groot, Jacinta Anna Lucke, Rosalinde A. L. Smits, Santiago Castejon-Hernandez, Lluis Llauger, Sira Aguiló Mir, Miguel Sánchez Ortiz, Eduardo Enrique Padilla, Santiago Cotobal Rodeles, Wojciech Rojewski-Rojas, Davide Fadini, Natalie Sabrina Jegerlehner, Christian Hans Nickel, Sara Rezzonico, Enrico Carlo Zucconi, Sumeyye Cakmak, Huseyin Avni Demir, Zerrin Defne Dündar, Ramazan Güven, Mehmet Akif Karamercan, Ozgur Sogut, Ismail Tayfur, James Alexander Adams, Janice Bernardo, Leanne Brown, Joel Burton, Matthew James Butler, Renate Isabelle Claassen, Francesca Compton, Jamie G. Cooper, Ruth Heyes, Sally Ko, Calvin John Lightbody, Jane A. H. Masoli, Stephen Thomas Gerard McKenzie, David Mawhinney, Nicola Jayne Moultrie, Angeline Price, Rajendra Raman, Lauren Heather Rothwell, Ravishankar Prabhakar Shashikala, Erica Jane Smith, Vittoria Sorice, James D. van Oppen, James Michael Wallace, Tom Young, Ana Benvin, Edita Breški, Alda Ćefo, Dijana Dumić, Rea Ferenac, Ivanka Jurica, Marinka Otočan, Petra Šverko Zinaić, Bénédicte Clement, Laurent Jacquin, Blandine Royer, Stefanie Irmgard Apfelbacher, Sofia Bezati, Sofia Gkarmiri, Christina V. Kaltsidou, George Klonos, Zoi Korka, Afroditi Koufogianni, Vasileios Mavros, Adamantia Nano, Angelos Ntousopoulos, Nikolaos Papadopoulos, Rakel Sason, Sofia-Chrysovalantou Zagalioti, Ingibjörg Hjaltadottir, Ingibjörg Sigurþórsdóttir, Sigrun Sunna Skuladottir, Thordis Thorsteinsdottir, Deirdre Breslin, Colm Patrick Byrne, Anita Dolan, Olivia Harte, Durriya Kazi, Aoife McCarthy, Shane Stephen McMillan, Dineo Ntesang Moiloa, Íde Louise O’Shaughnessy, Vinny Ramiah, Susan Williams, Tommaso Giani, Elena Levati, Rossella Montenero, Andrea Russo, Sara Salini, Bianca van den Berg, Anja Martine Booijen, Ozcan Sir, Anne Elisabeth Vermeulen, Michèle Anna ter Voert, Alicia C. Alvarez-Galarraga, Youcef Azeli, Rocío García-Gutiérrez Gómez, Rebeca González González, Dayris Lizardo, Marta López Pérez, Coral Núñez Madan, Jesus Ángel Medina, Javier Sierra Moreno, Erika Vanessa Bolívar Patiño, David Martín-Crespo Posada, Irene Cabrera Rodrigo, Catherine Franca Vitucci, Marco Ballinari, Thomas Dreher, Leone Gianinazzi, Tanguy Espejo, Wolf E. Hautz, Sara Rezzonico, Burcu Bayramoğlu, Sumeyye Cakmak, Burhan Comruk, Tuba Dogan, Fulya Köse, Thomas Paul Allen, Robert Ardley, Claire Marie Beith, Keith Alan Boath, Hannah Louise Britton, Marion Madeleine Françoise Campbell, Jonathon Capel, Conall Catney, Suzanne Clements, Brigid Pauline Collins, Francesca Compton, Alison Cook, Emma Jane Cosgriff, Tina Coventry, Nancileigh Doyle, Zoe Evans, Toluwalase Abdulrazak Fasina, John Francis Ferrick, Gail Mclaughlin Fleming, Caroline Gallagher, Mark Golden, Darshan Gorania, Lynn Glass, Hannah Greenlees, Zara Patricia Haddock, Ruth Harris, Carol Hollas, Amy Hunter, Claire Ingham, Shirley Sau Yin Ip, Jacqueline Anne James, Christopher Kenenden, Gabrielle Elizabeth Jenkinson, Emma Lee, Sophie Amelia Lovick, Margaret McFadden, Roisin McGovern, Jasmine Medhora, Farah Merchant, Srishti Mishra, Gayle Betsy Moreland, Subha Narayanasamy, Amy Rebecca Neal, Emma Louise Nicholls, Mariam Turkey Omar, Noleen Osborne, Favour Oghenevwaire Oteme, Jemma Pearson, Robert Price, Monika Sajan, Loveleen Kaur Sandhu, Harriet Scott-Murfitt, Beth Sealey, Eleanor Paige Sharp, Benjamin Andrew Charles Spowage-Delaney, Fiona Stephen, Lynn Stevenson, Ian Tyrrell, Chukwunonso Kalu Ukoh, Rebekah Walsh, Alice May Watson, June Elizabeth Cowan Whiteford, Corinne Allston-Reeve, Thomas James Barson, Margherita Grotzkyj Giorgi, Yasmin L. Godhania, Vicki Inchley, Evgeny Mirkes, Sajid Rahman

**Affiliations:** Centre for Urgent and Emergency Care Research, University of Sheffield, Sheffield, S1 4DA UK

**Keywords:** Frailty, Emergency care, Geriatrics

## Abstract

**Introduction:**

Current emergency care systems are not optimized to respond to multiple and complex problems associated with frailty. Services may require reconfiguration to effectively deliver comprehensive frailty care, yet its prevalence and variation are poorly understood. This study primarily determined the prevalence of frailty among older people attending emergency care.

**Methods:**

This cross-sectional study used a flash mob approach to collect observational European emergency care data over a 24-h period (04 July 2023). Sites were identified through the European Task Force for Geriatric Emergency Medicine collaboration and social media. Data were collected for all individuals aged 65 + who attended emergency care, and for all adults aged 18 + at a subset of sites. Variables included demographics, Clinical Frailty Scale (CFS), vital signs, and disposition. European and national frailty prevalence was determined with proportions with each CFS level and with dichotomized CFS 5 + (mild or more severe frailty).

**Results:**

Sixty-two sites in fourteen European countries recruited five thousand seven hundred eighty-five individuals. 40% of 3479 older people had at least mild frailty, with countries ranging from 26 to 51%. They had median age 77 (IQR, 13) years and 53% were female. Across 22 sites observing all adult attenders, older people living with frailty comprised 14%.

**Conclusion:**

40% of older people using European emergency care had CFS 5 + . Frailty prevalence varied widely among European care systems. These differences likely reflected entrance selection and provide windows of opportunity for system configuration and workforce planning.

**Supplementary Information:**

The online version contains supplementary material available at 10.1007/s41999-023-00926-3.

## Key summary points

**Aim:**

To determine the prevalence of frailty among older people attending emergency care.

**Findings:**

Across 14 European countries, 40% of older people using emergency care were living with at least mild frailty. 14% of all adult users were older people with frailty.

**Message:**

The high prevalence of frailty in emergency care indicates the need to accordingly configure healthcare systems and plan workforces.

**Supplementary Information:**

The online version contains supplementary material available at 10.1007/s41999-023-00926-3.

## Introduction

The core tenet of geriatric emergency medicine is frailty-attuned, holistic assessment [1, 2]. This multidimensional, multidisciplinary approach should culminate in shared decision-making [3, 4]. Current emergency care systems are not designed to deliver multidimensional care at scale and are instead modeled to rapidly deliver interventions for single and specific injuries or illnesses rather than evaluating multiple and complex problems [5, 6].

Healthcare service models across Europe are being reconfigured to better provide for the needs of older people living with frailty. However, the scale of response required is poorly understood as there is sparse objective evidence for the prevalence of frailty among users of unscheduled healthcare and its variation between settings. Variation in emergency department frailty prevalence will be heavily influenced by local and national factors, and its study would provide insights into the necessary organization of social care, primary care (general practice), and prehospital care services. Those service models already incorporating frailty-attuned practices are widely heterogeneous [5]. Recognition and evaluation of frailty prevalence and variation could further inform the selection, development, and optimization of future service models, while understanding its frequency among all users of emergency care may inform educational curricula and workforce planning.

There has been no previous survey across multiple European countries using a uniform method of data collection with a standardized measure of frailty. The FEED study used the Clinical Frailty Scale (CFS) which has validity with mortality, admission rates, and lengths of stay [7, 8]. There are alternative measures of frailty and there is no consensus on their administration; however, the CFS has good metric reliability and is already recommended for systematic administration in some health systems [9, 10].

The primary objective of the Frailty in European Emergency Departments (FEED) study was to report the prevalence and variation of frailty among emergency care users aged 65 + across Europe.

## Methods

FEED was a project orchestrated by the European Taskforce on Geriatric Emergency Medicine (ETGEM), which exists to promote, champion, and pioneer high-quality care for older people [11]. ETGEM is a collaboration between special interest groups of the European Geriatric Medicine Society (EuGMS) and the European Society for Emergency Medicine (EUSEM). This paper describes the core findings of the observational study focusing on the prevalence of frailty among older people using emergency care. In addition, this paper presents the proportion of older people living with frailty among all adult attenders to a subset of sites and describes associations of frailty with immediate emergency care outcomes.

### Design and settings

This study used a flash mob approach over one 24-h period. Flash mob studies engage many collaborators to gather a large volume of observational data from many sites in a short period [12–14]. Emergency departments were invited to participate using snowball sampling throughout Europe via mailing lists (ETGEM), research networks (European Geriatric Medicine Society and European Society for Emergency Medicine), and social media. A site coordinator was appointed at each participating ED. The manuscript was prepared following the STROBE guidelines for reporting cross-sectional studies [15].

### Data collection

Observational data were collected for all patients aged 65 + who attended (registered) at the participating departments during a 24-h period starting from midnight to 0800 h on Tuesday 04 July 2023. To determine the proportion of older adults with frailty among all emergency care users, those sites using electronic healthcare records also submitted data for all attenders aged 18 + .

Data variables were routinely collected as part of standard emergency care and included individuals’ age, Clinical Frailty Scale version 2.0 (CFS) [7], sex, ethnic group coded into UK Office for National Statistics categories [16], living arrangement including receipt of social care, mode of arrival to the ED, initial vital signs and NEWS2 score, ED arrival and departure times, use of resuscitation areas, and ED disposition outcome. Clinical teams were signposted to online training resources on CFS administration [17, 18]. These were not mandated for study participation. Data were collected by site coordinators and their appointed teams using REDCap or sites’ electronic health records.

### Statistical analysis

Data were examined for normality using graphical and Shapiro–Wilk methods. Normally distributed variables were reported as mean with standard deviation and skewed variables as median with interquartile range. Data were processed and analyzed using R with the packages choroplethr, ggplot2, lubridate, patchwork, and tidyverse [19].

### Frailty prevalence and variation

Frailty distribution was calculated at the pooled European level as the proportion of ED users aged 65 + with each CFS level and was dichotomized at the CFS = 5 threshold [20]. Variation in prevalence between countries was described as proportions and assessed for significance using the Kruskal–Wallis test.

The potential impact of missing CFS data and the presence of selection bias were assessed by analyzing complete and missing CFS records for differences in age, sex, ethnic group, mode of presentation, and acuity (national early warning score 2 (NEWS2) and use of resuscitation area) using logistic regression and Chi-square tests. The proportion of older people (aged 65 +) among all adult attenders (aged 18 +) was reported using data collected at those sites with electronic healthcare records.

### Frailty associations with emergency care outcomes

Frailty prevalence at the site level was compared using Spearman’s correlation with sites’ total daily attendances (only sites including all adults aged 18 +), site staffing levels, and site median ED lengths of stay.

At the individual level, Kruskal–Wallis and Chi-square tests were used to assess associations between older people’s frailty scores with demographic (age, sex, ethnic group, living arrangement) and emergency attendance factors (time and mode of arrival, ED length of stay compared to the site median, initial vital signs with NEWS2, use of the resuscitation room, and outcome of attendance including death and admission).

### Ethics and regulatory approval

All data were considered fully anonymized at the point of transfer. The study received ethical approval for data processing and the described analyses (University of Leicester ref 39,346). Site coordinators obtained further approvals for participation where required by local and national policies and legislation. Participants were consented for observation only where required by local and national approvals; in most cases, this requirement was waived for the collection of anonymized routine data. The protocol was deposited online (https://dx.doi.org/10.17504/protocols.io.ewov1ok97lr2/v1).

## Results

Sixty-two sites from fourteen countries participated in data collection (Supplementary material 1). These were in hospitals with 20-2659 inpatient beds and 8-278 ED trolley spaces. ED daily throughput ranged from 0.1 to 4.8 attendances per trolley space (median: 1.5). Twenty-two sites submitted age distributions for all adult (aged 18 +) patients. These sites had attendance totals over the 24-h period ranging from 29 (Radboud University Medical Centre) to 416 (Leicester Royal Infirmary), with median 172 (IQR, 178).

In total, data were collected for 5,785 individuals of whom 3,479 (60%) were aged 65 + . These people had median age 77 (IQR, 13) years and 53% were female. The CFS was missing for 9% older people, with no patterns of missingness identified (Supplementary material 2).

### Frailty prevalence and variation

**Fig. 1:**
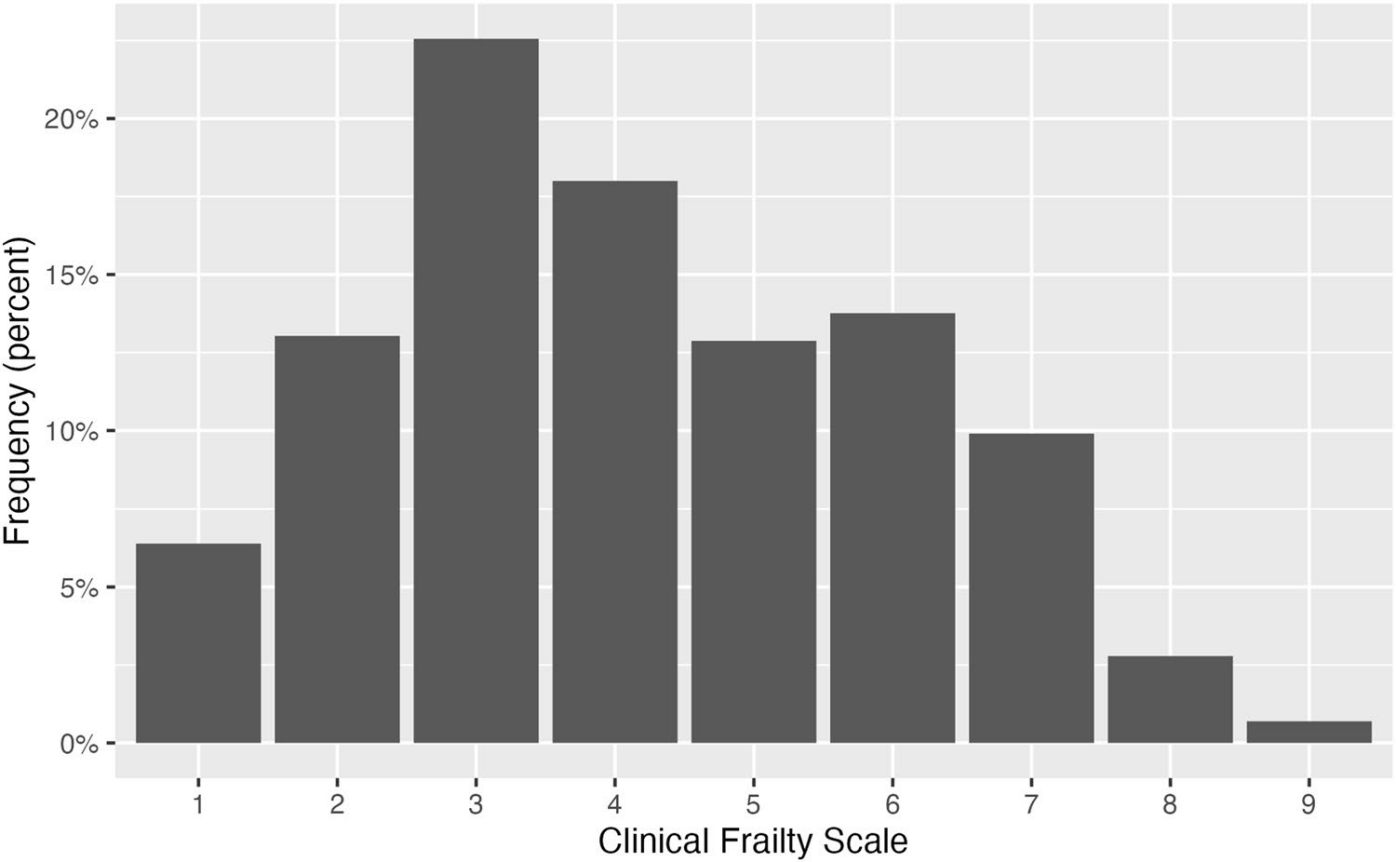
Distribution of Clinical Frailty Scale (all sites, individuals 65 +)

Among patients aged 65 + , 1265 (40% of complete observations) were living with mild or more severe frailty defined by CFS 5 + (Fig. 1). Median age increased with CFS = 1 (71 years) to CFS = 8 (85 years).

The country-level prevalence of frailty among older ED attenders had significant variation (*p* < 0.001). Prevalence ranged from 26% (Netherlands) to 51% (Switzerland) (Table 1).

**Table 1:** National-level frailty prevalence

Country	Sites	Individuals 65 +		Aged 65 + , CFS 5 +		
		N	CFS 5 + , *n* (%)	Admitted, %	Mortality, %	Median ED-LOS, hrs
Netherlands	4	97	25 (26)	40	4	3.6
Czech Republic	1	21	6 (29)	33	0	4.3
Croatia	1	64	22 (34)	36	0	4
Italy	1	73	25 (34)	76	4	55.4
France	4	188	66 (35)	56	2	7.8
Spain	7	277	98 (35)	42	2	5.6
Iceland	1	48	18 (38)	50	0	23.8
Ireland	5	166	65 (39)	72	0	16.4
Hungary	1	47	19 (40)	68	0	12.9
Greece	3	298	121 (41)	64	1	2.9
Turkey	7	514	209 (41)	33	1	4.1
United Kingdom	21	1197	505 (42)	66	1	5.9
Germany	1	68	33 (49)	88	0	3.6
Switzerland	5	103	53 (51)	64	0	4.6

Within the subset of 22 sites reporting data for all adult attenders, 35% of patients were aged 65 + . Correspondingly 14% adult users of emergency care were older people living with frailty.

### Frailty associations with emergency care outcomes

Admission rates increased with CFS, and there was more variation (a broader range) in the lowest (CFS 1 and 2) and highest (CFS 8 and 9) frailty levels. Higher frailty prevalence did not correlate with sites’ total daily attendances, staffing levels per ED space, or median ED lengths of stay.

At the individual level, higher CFS scores up to very severe frailty were associated with increasing age (*p* < 0.001) and receipt of social care (*p* < 0.001) (Table 2). There was no association with having non-white ethnic group (*p* = 0.377). Increasing CFS was associated with higher initial NEWS2 and more frequent use of the resuscitation area. Older people living with more severe frailty had more frequent admissions to hospital and deaths while in the ED. Length of ED stay did not vary substantially between CFS levels.

**Table 2:** Attendance characteristics by CFS level

CFS	Median age	Non-white, %	Receiving social care, %	Median NEWS2	Resuscitation area, %	Admitted, %	Mortality, %	Median ED-LOS, h
1	71	7	0	1	5	29	0	4.4
2	72	5	1	1	5	33	0	3.9
3	74	4	2	1	5	35	0	4.2
4	77	4	9	1	8	43	0	5
5	80	4	19	1	6	52	0	5.2
6	82	5	37	2	10	57	0	5.8
7	84	3	67	3	10	62	2	5.6
8	85	5	69	5	25	67	5	5.3
9	78	0	50	8	32	73	0	4.7

## Discussion

The FEED study was the largest cross-sectional evaluation of frailty in emergency care settings and broadly represents the European population. Frailty was prevalent among emergency care users in Europe, present in 40% attenders aged 65 + . Of all adult emergency care users, 14% were older people living with frailty. This has profound implications, indicating ongoing need for service model reconfiguration and workforce planning to deliver effective geriatric emergency care.

Around 10% of community-dwelling older people live with frailty, but it is observed far more frequently in hospital settings [21]. The overall European prevalence observed here in emergency care (40%) was very similar to a pooled prevalence reported in a systematic review of hospitalized older people (41%) [22]. That systematic review identified much broader variation of prevalence (5–93% older people across different ward settings) than that among older emergency care users here (26–51% across sixty-two sites). National frailty prevalence observed here was similar to previous single-center emergency care reports from Ireland (42% vs 29–60% [23, 24]) and England (42% vs 55% [8]), but lower in The Netherlands (26% vs 44% [25]) indicating possible site selection bias.

Emergency care frailty prevalence varied significantly between countries. Study participation spanned many healthcare systems and operating models. These were known to be heterogeneous in nature and are expected to have influenced the frailty prevalence observed. The large differences in median ED lengths of stay among older people living with frailty (3–55 h) demonstrate national differences in delivery and operating models of emergency and acute care. Variations in healthcare services and practices are further observed with the rates of admission for older people living with frailty ranging from 33% (Czech Republic and Turkey) to 88% (Germany). It is important to note that participating sites in certain countries (for example, The Netherlands) were predominantly specialized tertiary-level centers and may have seen attendances by different patient groups to those attending secondary-level emergency departments. However, the patterns observed here may reflect availability of community-based primary care services, emergency department ‘gate-keeping’ systems, and resourcing of inpatient admission beds (for example, Turkey compared to The Netherlands) [26].

Therefore, while the principal tenets of practice in geriatric emergency medicine may be transferrable, there is unlikely to be one single generic service model which suits all settings. There is an ongoing need for sites and health systems to generate and appraise evidence applicable to their specific situation. This information can then be used to determine optimal configuration of frailty-attuned services [27].

Recently established research priorities alluded to the limited evidence base informing emergency healthcare for older people living with frailty [28–31]. Current geriatric emergency care pathways and guidelines vary widely in design and nature [5]. These are often based on evidence in which people with frailty were poorly represented, and therefore specific research focus is required on identifying, defining, and evaluating interventions for this substantial group.

The observed prevalence of frailty is at odds with its scant representation in emergency medicine and nursing curricula. As older people living with frailty represent a substantial proportion of attenders to emergency care, healthcare professionals in these settings must possess the attitudes and competences necessary to provide optimal care [32]. High-quality geriatric emergency care requires skilled multidisciplinary collaboration [2, 4]. Healthcare systems must accordingly plan for workforce recruitment, training, and retention. Many service interventions to date have focused on reducing ED attendances and conveyances, and yet the frailty prevalence remains high and inevitably crises will still occur; there may be a need to redefine and transgress traditional boundaries between communities and hospitals to optimize the continuity of care.

Frailty confers additional risk to people using emergency care, evidenced here by longer stays, more frequent admissions, and higher mortality. These are the core outcome measures of emergency care, but may be most suited as metrics of flow and process through services and may have limited meaningfulness at the individual level [33]. A higher proportion of people living with more severe frailty received care in the resuscitation room setting. This may have reflected service inability to fulfill healthcare needs in major areas, or may have been a manifestation of cultural or legislative perspectives and competence in recognizing and appropriately managing intervention futility [34]. Redoubled efforts are required to tailor healthcare operating models, service improvement, and outcome measurement to individuals in accordance with the principles of person-centered and comprehensive geriatric care.

Our recruitment approach conferred the likelihood of over-representing those sites already highly engaged in geriatric emergency medicine delivery and improvement. The United Kingdom was over-represented while six countries had only one participating center. Sites’ participation may have been influenced by existing professional interests in frailty, and it is therefore possible that representation here is of hospitals with better-established frailty services.

This study aimed to collect the CFS from all attenders aged 65 + , and yet 9% records had missing data. No patterns of missingness were apparent based on hypothesized demographic and acuity factors. A more detailed evaluation of concordance with CFS screening will be reported separately.

We selected the CFS as a frailty measure in part due to its wide use in routine emergency care data. Ongoing controversy is acknowledged regarding frailty quantification in younger populations and in people living with stable disabilities, for whom the CFS is not validated [35].

## Conclusion

In this cross-sectional observational study of emergency care spanning 62 sites in 14 European countries, 40% of older people (65 +) were living with frailty. Frailty prevalence varied between countries (26–51%). It is important that emergency services are adapted and equipped to provide multidisciplinary care for this group of patients who often have complex health and care needs.

### Supplementary Information

Below is the link to the electronic supplementary material.

Supplementary file1 (DOCX 34 KB)

## Data Availability

The dataset generated and analysed during this study is not publicly available.
