# Supplementary material for: Prevalence of Frailty in European Emergency Departments (FEED): an international flash mob study
Source: Eur Geriatr Med. 2024 Feb 10;15(2):463–70. doi: 10.1007/s41999-023-00926-3 (PMC10997678; doi:10.1007/s41999-023-00926-3)
Supplement: Supplementary file 1 — Supplementary file1 (DOCX 34 KB) [file 41999_2023_926_MOESM1_ESM.docx]

# Supplementary material 1

## Characteristics of the participating sites

| Site | Hospital size (inpatient beds) | ED size (trolley spaces) | Rostered ED nurses overnight | Rostered ED doctors overnight | Age group returned | Attendances in observation period | |
| --- | --- | --- | --- | --- | --- | --- | --- |
|  |  |  |  |  |  | All adults | 65+ |
| Aberdeen Royal Infirmary | NA | 27 | 11 | 6 | All adults | 172 | 48 |
| AHEPA University Hospital | 663 | 57 | 14 | 17 | All adults | 258 | 95 |
| Althaia Xarxa Assistencial Universitària de Manresa | 400 | 40 | 12 | 2 | Older people |  | 47 |
| Attikon University Hospital | 600 | 45 | 10 | 8 | All adults | 339 | 102 |
| Başakşehir Çam and Sakura City Hospital | 2650 | 278 | 40 | 20 | Older people |  | 106 |
| Bellinzona Hospital | NA | NA | NA | NA | Older people |  | 14 |
| Cambridge University Hospitals NHS Foundation Trust | 1037 | 69 | 24 | 13 | All adults | 312 | 102 |
| Chesterfield Royal Hospital | 682 | 59 | 13 | 5 | All adults | 253 | 93 |
| Clinical Hospital Center Rijeka | 1069 | 30 | 10 | 10 | Older people |  | 64 |
| Daisy Hill | 147 | 15 | 8 | 2 | Older people |  | 30 |
| East Suffolk and North Essex | NA | 16 | NA | 7 | Older people |  | 70 |
| Fakulty Hospital Královské Vinohrady | 1100 | 20 | 6 | 3 | All adults | 36 | 21 |
| Fondazione Policlinico Universitario Agostino Gemelli IRCCS | 1500 | 150 | 6 | 3 | Older people |  | 73 |
| Fundació Sanitària Mollet | 20 | 80 | 3 | 3 | Older people |  | 10 |
| Gazi University Hospital | 1004 | 34 | 10 | 9 | Older people |  | 34 |
| Hospices Civils de Lyon (a) | NA | NA | NA | NA | Older people |  | 35 |
| Hospices Civils de Lyon (b) | NA | NA | NA | NA | Older people |  | 23 |
| Hospital Clinic Barcelona | 819 | 125 | 23 | 20 | Older people |  | 98 |
| Hospital de Terrassa | 521 | 62 | 10 | 7 | Older people |  | 42 |
| Hospital Obispo Polanco | NA | NA | NA | NA | Older people |  | 18 |
| Hospital Severo Ochoa | NA | NA | NA | NA | Older people |  | 55 |
| Hospital Universitario Sant Joan de Reus | 300 | 54 | 12 | 4 | Older people |  | 26 |
| Inselspital, Bern University Hospital | 1000 | 28 | 9 | 6 | Older people |  | 20 |
| Karamanoğlu Mehmetbey Üniversity Hospital | 400 | 20 | 4 | 3 | Older people |  | 44 |
| Kettering General Hospital | 540 | 48 | 21 | 7 | Older people |  | 88 |
| Klinikum Nurnberg | 2150 | 45 | 10 | 7 | Older people |  | 72 |
| Landspitali / National University Hospital of Iceland | 628 | 52 | 11 | 4 | Older people |  | 48 |
| Leicester Royal Infirmary | 1991 | 92 | 35 | 16 | All adults | 416 | 161 |
| Leiden University Medical Centre | 650 | 16 | 5 | 2 | Older people |  | 21 |
| Letterkenny University Hospital | 302 | 16 | 10 | 4 | All adults | 87 | 30 |
| Mater Misericordiae University Hospital | 719 | 30 | 18 | 4 | All adults | 164 | 50 |
| Midland Regional Hospital Tullamore | 195 | 21 | 9 | 2 | All adults | 73 | 29 |
| Necmettin Erbakan University Meram Medical Faculty | 1350 | 49 | 12 | 12 | Older people |  | 41 |
| Ospedale di Lugano | 20 | NA | 5 | 5 | Older people |  | 26 |
| Ospedale Regionale di Mendrisio | 130 | 8 | 2 | 2 | Older people |  | 12 |
| Princess Alexandra Hospital | 414 | 24 | 16 | 6 | Older people |  | 68 |
| Radboud University Medical Centre | NA | NA | NA | NA | All adults | 29 | 9 |
| Royal Berkshire Hospital | NA | NA | NA | NA | All adults | 203 | 62 |
| Royal Devon University Healthcare | 672 | 20 | 13 | 5 | Older people |  | 54 |
| Royal Stoke UHNM | 1200 | 74 | 24 | 9 | All adults | 248 | 99 |
| Royal Surrey County Hospital | 432 | 28 | 12 | 4 | Older people |  | 62 |
| Royal Victoria Hospital | 611 | 53 | 20 | 5 | Older people |  | 76 |
| Salford Royal | 700 | 40 | 19 | NA | Older people |  | 52 |
| Sancaktepe Sehit Prof. Dr. Ilhan Varank Training and Research Hospital | 1600 | 110 | 32 | 17 | Older people |  | 87 |
| Semmelweis University | 2593 | 30 | 14 | 8 | All adults | 112 | 47 |
| Shrewsbury and Telford Hospital | 700 | 74 | 20 | 6 | Older people |  | 102 |
| South West Acute Hospital | 210 | 18 | 8 | 2 | Older people |  | 18 |
| Spaarne Gasthuis | 393 | 35 | 6 | 2 | Older people |  | 78 |
| Stoke Mandeville Hospital | NA | 20 | 13 | 5 | Older people |  | 54 |
| Toulouse University Hospital (a) | NA | NA | NA | NA | All adults | 326 | 86 |
| University Hospital Basel | 550 | 43 | 9 | 6 | All adults | 108 | 36 |
| University Hospital Hairmyres | 498 | 21 | 9 | 2 | Older people |  | 48 |
| University Hospital Limerick | NA | NA | NA | NA | All adults | 78 | 44 |
| University Hospital Monklands | 430 | 21 | 7 | 4 | Older people |  | 56 |
| University Hospital of Tours | NA | NA | NA | NA | Older people |  | 44 |
| University Hospital Wishaw | 626 | 22 | 10 | 3 | Older people |  | 34 |
| University Medical Centre Groningen | 1339 | 27 | 4 | 1 | Older people |  | 26 |
| University of Health Sciences Haseki Training and Reseach Hospital | 800 | 65 | 12 | 8 | Older people |  | 70 |
| University of Health Sciences Sanliurfa Mehmet Akif Inan | 625 | 34 | 13 | 7 | Older people |  | 132 |
| Venizelion Hospital of Heraklion | 400 | 21 | 5 | 5 | All adults | 263 | 101 |
| Victoria Hospital Kirkcaldy | 450 | 25 | 8 | 4 | Older people |  | 57 |
| Warrington | 539 | 47 | 14 | 5 | All adults | 70 | 29 |

# Supplementary material 2

## Evaluation of CFS record missingness

| Variable | CFS complete n=3161 (91%) | CFS missing n=318 (9%) | Test for difference, p-value |
| --- | --- | --- | --- |
| Median age, years (IQR) | 77 (13) | 76 (13) | 0.324 |
| Proportion female, % | 53.2 | 52.2 | 0.850 |
| Proportion non-white, % | 4.3 | 3.1 | 0.730 |
| Proportion self-presenting, % | 42.8 | 40.3 | 0.502 |
| Median NEWS2 (IQR) | 1 (3) | 1 (2) | 0.928 |
| Proportion using resuscitation, % | 7.7 | 4.4 | 0.406 |
